# An extended data envelopment analysis for the decision-making

**DOI:** 10.1186/s13660-017-1502-0

**Published:** 2017-10-02

**Authors:** Xiao-Li Meng, Fu-Gui Shi

**Affiliations:** 1 School of Mathematics and Statistics, Beijing Institute of Technology, Beijing, 100081 China; 2 Beijing Key Laboratory on MCAACI, Beijing Institute of Technology, Beijing, 100081 China

**Keywords:** data envelopment analysis, sample standards, time series analysis, binary search tree, decision-making

## Abstract

Based on the CCR model, we propose an extended data envelopment analysis to evaluate the efficiency of decision making units with historical input and output data. The contributions of the work are threefold. First, the input and output data of the evaluated decision making unit are variable over time, and time series method is used to analyze and predict the data. Second, there are many sample decision making units, which are divided into several ordered sample standards in terms of production strategy, and the constraint condition consists of one of the sample standards. Furthermore, the efficiency is illustrated by considering the efficiency relationship between the evaluated decision making unit and sample decision making units from constraint condition. Third, to reduce the computation complexity, we introduce an algorithm based on the binary search tree in the model to choose the sample standard that has similar behavior with the evaluated decision making unit. Finally, we provide two numerical examples to illustrate the proposed model.

## Introduction

In conventional data envelopment analysis (DEA) models, such as CCR model named after Charnes et al. [1] and BCC model proposed by Banker et al. [2], the inputs and outputs are assumed to be precise. In addition, the constraint condition consists of the evaluated decision making units (DMUs).

In practical studies, the input and output data of the evaluated DMUs are frequently variable over multiple time periods (time series data), and it is important to analyze the change of efficiency over time. For example, in the evaluation of travel agencies, transportation, ticket price, accommodation, and labor are always regarded as the inputs, whereas profits and satisfaction of tourists are the outputs. The inputs and outputs are affected by various influential factors, such as the tourism policy, investment of infrastructure, level of starred hotel, annual per-capita income, and level of economic development. However, since the influential factors are variable over time, the inputs, outputs, and efficiencies of travel agencies are variable over time accordingly. Given the current upsurge in interest in DEA, it is surprising that the dynamic DEA attracts very little attention. The only methods we know of this area are Malmquist Productivity Index (MPI) and window analysis. MPI was originally proposed by Caves et al. [3] to estimate changes in the overall productivity growth of each DMU over a two-year period by calculating the efficiency value. To deal with the productivity changes of DMUs over time, Färe et al. [4] constructed a DEA-based MPI by combining the efficiency measurement of Farrell [5] with the productivity measurement of Caves et al. Window analysis, proposed by Charnes et al. [6], is adopted to overcome the constraint of limited DMUs and is a benefit to detect the tendency of DMUs over long period with large inputs and outputs. Since then, some improved approaches on the DEA-based MPI or window analysis have been proposed [7–17]. However, both the DEA-based MPI and window analysis models suffer from one shortcoming: they neglect predicting efficiency of the evaluated DMU.

In many practical evaluation problems, efficiency of every evaluated DMU in a particular period may not be contrasted with the evaluated DMUs, but rather with sample standards determined by manufacturing parameters. The purpose of the contrast is not only to evaluate efficiency, but also to locate the standard with which the evaluated DMU has similar behavior. For instance, there are many grade standards for the evaluation of travel agencies. Travel agencies from the same region can be evaluated by the same standards separately, and those from different regions should not be evaluated by the same standards because of regional disparities. The standards should be formulated by the regional parameters. Taking outbound tourism as an example, it is an important part for travel agency business in developed regions, but it may not be contained in the travel agency business in some developing regions. Clearly, it is unreasonable that the outbound tourism is included in input measures to evaluate the travel agencies from different regions, and then grade standards in different regions should be formulated in terms of different manufacturing parameters. With these preparations, we then could use different standards to evaluate the level of travel agencies. However, in the existing DEA models, the constraint condition consists of the evaluated DMUs. Furthermore, we categorize DEA models into two types. The first type is the DEA models where the DMU under evaluation is included in the constraint condition [18–39]. The second type is the DEA models where the DMU under evaluation is not included in the constraint condition. For example, Andersen and Petersen [40] developed the superefficiency DEA model, which is identical to the BCC model, except that the DMU under evaluation is not included in the constraint condition. Superefficiency DEA model has been fully explored and applied [41–44].

Without such considerations, scholars will not be tempted to invest the effort in analyzing and predicting the development trend of the DMUs by contrasting with grade standards. In fact, managers can analyze and predict the development trend of input and output data based on historical data and then determine the level by contrasting with sample standards. Furthermore, to maximize profit and ensure proper resource allocation management, efforts can be made through improving influential factors. Therefore, it is a scenario that is worth considering in this case.

The rest of this paper is organized unfolded as follows. Section 2 introduces the CCR model and the time series method. In Section 3, an extended DEA model is proposed. In Section 4, the relationship between DEA efficiency and the production frontier is illustrated. In Section 5, the algorithm to determine sample standards is described. In Section 6, two numerical examples are given to illustrate the proposed model. At the end of the paper, some conclusions are drawn.

## Preliminaries

### CCR model

As a most frequently used DEA model, the CCR model (Charnes et al. [1]) supposes that there are *n* DMUs and that each DMU consumes the same input type and produces the same output type. Let *m* and $s'$ be the numbers of inputs and outputs, respectively. All inputs and outputs are assumed to be positive. The multiple inputs and multiple outputs of each DMU are aggregated into a single virtual input and a single virtual output. The efficiency of the evaluated DMU is obtained as a ratio of its virtual output to its virtual input subject to the condition in which the ratio of each DMU is not greater than 1. The corresponding model is as follows:

1 $$ (\mathrm{CCR})\quad \textstyle\begin{cases} \max_{u,v} \frac{u^{T}y_{0}}{v^{T}x_{0}}, \\ \text{subject to} \\ \frac{u^{T}y_{j}}{v^{T}x_{j}} \le 1, \\ j = 1, \ldots,j_{0}, \ldots,n, \\ v \ge 0,\qquad v \ne 0, \\ u \ge 0,\qquad u \ne 0, \end{cases} $$

where $x_{j} = (x_{1j}, \ldots, x_{mj})^{T}$ and $y_{j} = (y_{1j}, \ldots, y_{s'j})^{T}$ are the input and output vectors of the *j*th DMU, DMU $j _{0}$ is the evaluated DMU, and *u* and *v* are the weight column vectors of outputs and inputs, respectively. The constraint condition consists of all the evaluated DMUs. By applying the Charnes-Cooper transformation (Charnes and Cooper [45]) in the model (1), the following equivalent linear model is obtained:

2 $$ (P_{\operatorname{CCR}} )\quad \textstyle\begin{cases} \max_{\mu} \mu^{T}y_{0}, \\ \text{subject to} \\ w^{T}x_{j} - \mu^{T}y_{j} \ge 0, \\ j = 1, \ldots, j_{0}, \ldots,n, \\ w^{T}x_{0} = 1, \\ w \ge 0, \\ \mu \ge 0,\qquad \mu \ne 0. \end{cases} $$

The optimal objective values of models (1) and (2) fall into the range of (0, 1]. The relationship between DEA efficiency and the optimal objective value (Cooper et al. [46]) can be obtained as follows.

#### Definition 1

DEA efficient

If the optimal objective value of the evaluated DMU is equal to 1 and there is at least one optimal solution in which the optimal weight vectors of inputs and outputs are greater than 0, then the evaluated DMU is DEA efficient.

#### Definition 2

weak DEA efficient

If the optimal objective value of the evaluated DMU is equal to 1 and there is no optimal solution in which the optimal weight vectors of inputs and outputs are greater than 0, then the evaluated DMU is weak DEA efficient.

#### Definition 3

DEA inefficient

If the optimal objective value of the evaluated DMU is less than 1, then the evaluated DMU is DEA inefficient.

### Time series method

A discrete ordered set of observed data that changes over time is called a time series and denoted as $y(t) = \{ y(t_{1}), y(t_{2}), \ldots,y(t_{i}), \ldots \}$, where $y(t_{i})$ is the observed data at the moment $t _{i}$. Time series can be divided into nonparametric and parametric models. The nonparametric model estimates the covariance or the spectrum without assuming that the process has a particular structure. By contrast, the parametric model assumes that the underlying stationary stochastic process has a certain structure. The time series model is used to extract meaningful statistic and other characteristics of the observed data and then to predict the development trend. It is usually composed of three parts, namely,

3 $$ Y(t) = f(t) + p(t) + X(t) , $$

where $f(t)$ is the trend term, which reflects the changing trend of $Y(t)$, $p(t)$ is the periodic term, reflecting the cyclical change of $Y(t)$, and $X(t)$ is the stochastic term, which reflects the influence of random factors of $Y(t)$. Here we assume that $X(t)$ is a normal stationary stochastic process (Chatfield [47], Gershenfeld [48]).

## An extended DEA model

In this section, based on the fundamental CCR model,we propose an extended DEA model. In the model, the input and output data of the evaluated DMUs are predicted by the time series method based on the historical data. The constraint condition consists of one of the sample standards determined by the production strategy. There are many sample DMUs, which are further divided into several ordered sample standards in terms of manufacturing parameters. Moreover, sample DMUs in the same standard have similar behavior. It is important to stress here that the evaluated DMU does not belong to the set of sample DMUs. The extended DEA model is as follows:

4 $$ \textstyle\begin{cases} \max_{u, v} \frac{u^{T}y_{E}(t)}{v^{T}x_{E}(t)}, \\ \text{subject to} \\ \frac{u^{T}\bar{y}_{kh}(s)}{v^{T}\bar{x}_{kh}(s)} \le 1, \\ k = 1, \ldots,\bar{m},\qquad h = 1, \ldots,\bar{n}_{k}, \\ v \ge 0,\qquad v \ne 0, \\ u \ge 0,\qquad u \ne 0, \end{cases} $$

where $x_{E}(t)$ and $y_{E}(t)$ are the input and output vectors of the evaluated DMU at the moment *t*, every element of $x_{E}(t)$ and $y_{E}(t)$ is nonnegative, and $\bar{x}_{kh}(s)$ and $\bar{y}_{kh}(s)$, which are determined in terms of the manufacturing parameter *s*, are the vectors of inputs and outputs of the *h*th sample DMU in the *k*th standard. There are *m̄* standards, and the *k*th ($k = 1, \ldots,\bar{m}$) standard is composed of $\bar{n}_{k}$ sample DMUs. The efficiency of the evaluated DMU is obtained from the maximum of the ratio of weighted outputs to inputs, and the ratio is less than or equal to 1 for every sample DMU from the standard regarded as constraint condition. The corresponding linear programming model is

5 $$ \textstyle\begin{cases} \max_{\mu} \mu^{T}y_{E}(t), \\ \text{subject to} \\ w^{T}\bar{x}_{kh}(s) - \mu^{T}\bar{y}_{kh}(s) \ge 0, \\ k = 1, \ldots,\bar{m},\qquad h = 1, \ldots,\bar{n}_{k}, \\ w^{T}x_{E}(t) = 1, \\ w \ge 0,\qquad w \ne 0, \\ \mu \ge 0,\qquad \mu \ne 0. \end{cases} $$

It is easy to see that the evaluated DMU is not contained in the constraint condition. The optimal objective values of $\frac{u^{T}y_{E}(t)}{v^{T}x_{E}(t)}$ and $\mu^{T}y_{E}(t)$ in models (4) and (5) vary in $(0, + \infty)$. The superefficiency definition of the proposed model is given as follows.

### Definition 4

DEA superefficient

An evaluated DMU is DEA superefficient if its optimal objective value is higher than 1 and there is at least one optimal solution in which the optimal weight vectors of inputs and outputs are greater than 0.

To determine the efficiency of the evaluated DMUs in the proposed model, the following theorems are given by considering the relationship between DEA efficiency and the optimal objective value.

### Theorem 1

*If the evaluated DMU is DEA superefficient by the* *kth standard*, *then the optimal objective value is greater than* 1.

### Theorem 2

*The evaluated DMU is DEA efficient by all the combinations of sample DMUs in the* *kth standard if and only if there exists an optimal objective value that is equal to* 1 *and the optimal weight vectors of inputs and outputs are greater than* 0.

### Theorem 3

*The evaluated DMU is weak DEA efficient by the* *kth standard if and only if the optimal objective value is equal to* 1 *and there does not exist any optimal solution in which the optimal weight vectors of inputs and outputs are greater than* 0.

### Theorem 4

*The evaluated DMU is DEA inefficient by all the combinations of sample DMUs in the* *kth standard if and only if all optimal objective values are less than* 1.

## The relationship between DEA efficiency and the production frontier

In this section, we consider the case of two inputs and a single output to show the relationship between DEA efficiency and the production frontier. DEA efficiency is independent of the change of inputs and output by the same proportion, so we can change the inputs and output in the same proportion for each DMU until the output data of the evaluated DMUs and sample DMUs are equal. Next, the coordinate system is established with input 1 and input 2 as the *x* and *y* coordinate axes. For the DMU in the coordinate system, the closer it gets to the coordinate origin, the higher efficiency will be.

### DEA efficiency and the production frontier in the conventional DEA models

**Figure 1:**
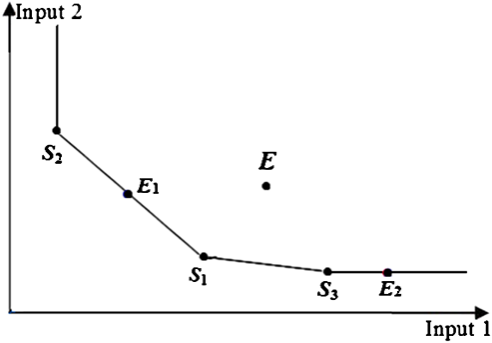
**The DEA efficiency in CCR model.**

In the CCR model, the constraint condition consists of all the DMUs, and the production frontier is spanned by efficient DMUs and weak efficient DMUs. As shown in Figure 1, the production frontier is spanned by DMUs $S _{1}$, $S _{2}$, $S _{3}$, $E _{1}$, and $E _{2}$. DMUs $S _{1}$, $S _{2}$, $S _{3}$, and $E _{1}$ are DEA efficient, DMU $E _{2}$ is weak DEA efficient, and DMU *E* is DEA inefficient.

**Figure 2:**
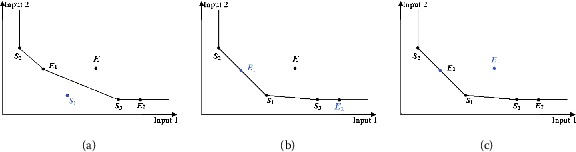
**The DEA efficiency in the superefficiency DEA.**

In the superefficiency model, the constraint condition consists of all the DMUs except the evaluated DMU, and the production frontier is spanned by all the corresponding DMUs without the DMU under evaluation. If the evaluated DMU is located on the weak production frontier, then it is weak efficient (Yu et al. [49], Wei et al. [50]). If the evaluated DMU is located on the efficient production frontier, then it is efficient (that is, there exist positive optimal weight vectors of inputs and outputs such that the efficiency of the evaluated DMU is equal to the efficiency of a certain sample DMUs and the optimal objective value is equal to 1 (Doyle and Green [51], Salo and Punkka [52]). If the evaluated DMU is located in the production possibility set but is not located on the production frontier, then it is inefficient. Otherwise, the evaluated DMU is superefficient. For example, the evaluated DMU $S _{1}$ is superefficient in Figure 2(a), the evaluated DMUs $E _{1}$ and $E _{2}$ are efficient and weak efficient, respectively, in Figure 2(b), and the evaluated DMU *E* is inefficient in Figure 2(c).

### DEA efficiency and the production frontier in the proposed model

Unlike conventional DEA models, in the proposed model, the constraint condition consists of one of the sample standards, and the production frontier is spanned by different combinations of sample DMUs from the constraint condition. To illustrate this, now we suppose that there are seven evaluated DMUs $E _{3}$-$E _{9}$ and that the *k*th standard is the constraint condition consisting of nine sample DMUs $S _{4}$-$S _{12}$.

**Figure 3:**
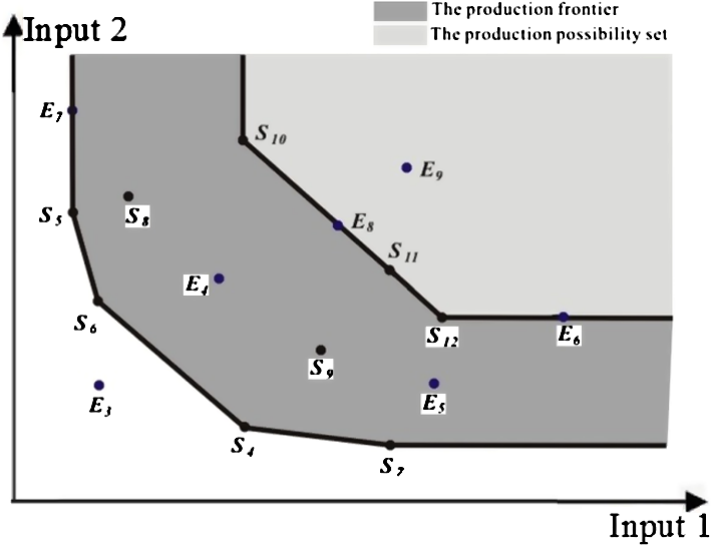
The DEA efficiency and production frontier in the proposed model.

The consequence of all the combinations of sample DMUs in the *k*th standard is easily understood in terms of Figure 3. The production frontier of sample DMUs $S _{4} $-$S _{12}$ is shown in the shaded portion. We can see that the most efficient production frontier is spanned by sample DMUs $S _{4}$-$S _{7}$, the least efficient production frontier is spanned by sample DMUs $S _{10}$-$S _{12}$, and the other production frontiers that are spanned by different combinations of sample DMUs $S _{4}$-$S _{12}$ are located between the most and least efficient production frontiers.

The evaluated DMU $E _{3}$ is closer to the coordinate origin than the most efficient production frontier, and then the efficiency of DMU $E _{3}$ is higher than that of every sample DMU from the constraint condition. In such a case, the constraint condition consists of all sample DMUs of the *k*th standard, the optimal objective value is greater than 1, and the evaluated DMU $E _{3}$ is DEA superefficient.

If the evaluated DMU is located between the most and least efficient production frontiers, then there is at least one optimal objective value equal to 1 for the evaluated DMU, such as DMU $E _{4}$, $E _{5}$, $E _{6}$, $E _{7}$, and $E _{8}$. Clearly, in Figure 3, it is easy to see that DMU $E _{4}$ is DEA superefficient by the least efficient production frontier $S _{10}$-$S _{12}$ and DEA inefficient by the most efficient production frontier $S _{4}$-$S _{7}$; then DMU $E _{4}$ is DEA efficient (i.e., the optimal objective value of the evaluated DMU $E _{4}$ is equal to 1, and the optimal weight vectors of inputs and outputs are greater than 0) by a combination of the *k*th standard. Similarly, there is an optimal objective value of DMU $E _{5}$ equal to 1, and the optimal weight vectors of inputs and outputs are greater than 0. In the following, we consider the evaluated DMU $E _{6}$, which is weak DEA efficient relative to the least efficient production frontier, and then there is an optimal objective value of the DMU $E _{6}$ equal to 1. A similar analysis applies to the evaluated DMU $E _{7}$; we can see that there is also an optimal objective value equal to 1. Finally, we take the evaluated DMU $E _{8}$ into account, it can be expressed by a linear combination of DMU $S _{10}$ and $S _{11}$, and thus it is DEA efficient by the least efficient production frontier, and there is an optimal objective value equal to 1. The evaluated DMU $E _{9}$ is located in the production possibility set of the least efficient production frontier but not located on the least efficient production frontier. Then DMU $E _{9}$ is DEA inefficient, and the optimal objective value is less than 1. In fact, in the proposed model, the determined production frontier is spanned by the difference between the production possibility sets of the most and least efficient production frontiers.

## Algorithm

Sample DMUs are divided into *m̄* ordered sample standards, and it is important to stress that *m̄* may be a very large value. In such a case, there will be high computation complexity if we locate the standard individually. Differently from the published works that address the problem of reducing computation complexity in DEA (e.g., Dulá [53], Dulá and Thrall [54]), we introduce the algorithm based on a binary search tree in the proposed model to determine the sample standard with which the evaluated DMU has similar behavior. If the evaluated DMU is superefficient by the *t*th standard, then the constraint condition should turn to the standard with higher efficiency. If the evaluated DMU is weak efficient or inefficient by the *t*th standard, then the constraint condition should turn to the standard with lower efficiency. Otherwise, the evaluated DMU is located in the *t*th standard, that is, the evaluated DMU has similar behavior with the *t*th standard. Let $[x]$ denote the greatest integer not greater than *x*. The algorithm is summarized as follows.

***Step*0:** Star with dividing the sample DMUs into *m̄* ordered sample standards. Let $t _{1}=1$, $t _{2}=\bar{m}$.
***Step*1:** Use the $t _{1}$th and $t _{2}$th standards to evaluate the evaluated DMU.
  If the evaluated DMU is DEA efficient by the $t _{1}$th (or $t _{2}$th) standard, then
  - Stop - the evaluated DMU has similar behavior with the $t _{1}$th (or $t _{2}$th) standard;
  If the evaluated DMU is DEA superefficient (or inefficient) by the $t _{1}$th and the $t _{2}$th standard, then
  - Stop - the evaluated DMU has not similar behavior with all the sample standards;
  Else
  - Turn to Step 2.
***Step*2:** If the evaluated DMU is DEA superefficient by the $t _{1}$th standard and inefficient by the $t _{2}$th standard, then
  - $t _{3} \leftarrow[(t _{1}+t _{2})/2]$
  - If the evaluated DMU is DEA inefficient by the $t _{3}$th standard, then
    - $t _{2} \leftarrow t _{3}$; Turn to Step 2;
  - If the evaluated DMU is DEA superefficient by the $t _{3}$th standard, then
    - $t _{1} \leftarrow t _{3}$; Turn to Step 2;
  - Else
    - Turn to Step 1.
  If the evaluated DMU is DEA superefficient by the $t _{1}$th standard and weak efficient by the $t _{2}$th standard, then
  - Stop - the evaluated DMU has similar behavior with the $(t _{2}-1)$th standard.
  If the evaluated DMU is DEA inefficient by the $t _{1}$th standard and superefficient by the $t _{2}$th standard, then
  - $t _{3} \leftarrow[(t _{1}+t _{2})/2]$
  - If the evaluated DMU is DEA superefficient by the $t _{3}$th standard, then
    - $t _{2} \leftarrow t _{3}$; Turn to Step 2;
  - If the evaluated DMU is DEA inefficient by the $t _{3}$th standard, then
    - $t _{1} \leftarrow t _{3}$; Turn to Step 2;
  - Else
    - Turn to Step 1.
  If the evaluated DMU is DEA weak efficient by the $t _{1}$th standard and superefficient by the $t _{2}$th standard, then
  - Stop - the evaluated DMU has similar behavior with the $(t _{1}+1)$th standard;

## Illustrative examples

In this section, we present two numerical examples to illustrate the proposed model. For simplicity, “sample DMU” will be abbreviated to “SDMU”.

### The first example

In this example, the data of the evaluated DMU in the last 23 years are provided in Table 1. There are 15 sample DMUs with two inputs and a single output listed in Table 2, and the sample DMUs $\mathrm{SDMU}_{i1}$, $\mathrm{SDMU}_{i2}$, and $\mathrm{SDMU}_{i3}$ ($i=1, 2, 3, 4, 5$) are located in the *i*th standard. By the proposed model the production status of the evaluated DMU in the 24th year is analyzed.

**Table 1:** **Production status in the last 23 years**

**Year**	**Input 1**	**Input 2**	**Output**	**Year**	**Input 1**	**Input 2**	**Output**
1	7.2	6.9	16.6	13	6.9	5.5	20.6
2	7.8	5.1	20.1	14	7.9	5.8	20.7
3	7.3	5.7	20.5	15	8.1	6.4	18.3
4	6.5	6.7	20.0	16	6.8	6.2	19.1
5	7.0	6.5	21.5	17	6.5	5.7	17.5
6	7.2	5.5	16.6	18	6.9	5.6	15.0
7	7.4	5.7	15.4	19	7.1	6.2	16.1
8	7.1	6.0	17.7	20	7.0	6.5	20.1
9	7.3	6.1	19.4	21	7.2	6.0	18.1
10	7.5	6.3	16.4	22	7.2	5.9	17.4
11	7.3	6.6	16.5	23	7.2	5.8	16.4
12	6.4	6.1	20.1

**Table 2:** **The sample standards**

	**Standard 1**			**Standard 2**			**Standard 3**			**Standard 4**			**Standard 5**		
	$\boldsymbol{\mathrm{SDMU}_{11}}$	$\boldsymbol{\mathrm{SDMU}_{12}}$	$\boldsymbol{\mathrm{SDMU}_{13}}$	$\boldsymbol{\mathrm{SDMU}_{21}}$	$\boldsymbol{\mathrm{SDMU}_{22}}$	$\boldsymbol{\mathrm{SDMU}_{23}}$	$\boldsymbol{\mathrm{SDMU}_{31}}$	$\boldsymbol{\mathrm{SDMU}_{32}}$	$\boldsymbol{\mathrm{SDMU}_{33}}$	$\boldsymbol{\mathrm{SDMU}_{41}}$	$\boldsymbol{\mathrm{SDMU}_{42}}$	$\boldsymbol{\mathrm{SDMU}_{43}}$	$\boldsymbol{\mathrm{SDMU}_{51}}$	$\boldsymbol{\mathrm{SDMU}_{52}}$	$\boldsymbol{\mathrm{SDMU}_{53}}$
Input1	1	3	2	1	3	2	1	3	2	1	3	2	1	3	2
Input2	2	1	2	2	1	2	2	1	2	2	1	2	2	1	2
Output	[0,1]	[0,2]	[0,3]	(1,2]	(2,3]	(3,4]	(2,3]	(3,4]	(4,5]	(3,4]	(4,5]	(5,6]	(4,5]	(5,6]	(6,7]

**Figure 4:**
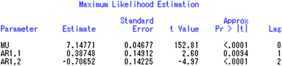
**Maximum likelihood estimation of Input 1.**

Firstly, time series method is used to analyze the inputs and outputs. In Figure 4, all the *p* values of *t*-statistics are less than 0.05, and then the original hypothesis whose parameter is 0 should be rejected, and the three unknown parameters are considered to be significant. The AR(2) model is suitable for fitting the Input 1 sequence, and the predicting equation is as follows:

$$x_{1}(t) = 7.14771 + 0.38748x_{1}(t - 1) - 0.70652x_{1}(t - 2). $$

Similarly, the predicting equations of Input 2 and Output are given respectively by

$$\begin{gathered} x_{2}(t) = 6.07144 - 0.72731x_{2}(t - 1), \\ y(t) = 18.17864 - 0.54212y(t - 1). \end{gathered} $$

The DEA model is as follows:

6 $$ \textstyle\begin{cases} \max_{\mu} \mu^{T} [ 18.179 - 0.542y(t - 1) ], \\ \text{subject to} \\ w^{T}\bar{x}_{kh} - \mu^{T}\bar{y}_{kh} \ge 0, \\ k = 1, \ldots,5;\qquad h = 1,2,3, \\ w_{1} [ 7.14771 + 0.38748x_{1}(t - 1) - 0.70652x_{1}(t - 2) ]\\ \quad {} + w_{2} [ 6.07144 - 0.72731x_{2}(t - 1) ] = 1, \\ w \ge 0,\qquad w \ne 0;\qquad \mu \ge 0,\qquad \mu \ne 0. \end{cases} $$

Secondly, the production status of the evaluated DMU in the 24th year is evaluated by the sample standards. Since the outputs of the constraint condition are interval values, it is impossible to calculate every value. Then the two endpoints of interval are defined as the pessimistic and optimistic values separately (Wang et al. [22]). For example, *a* is the pessimistic value, and *b* is the optimistic value in the interval $(a, b]$ or $[a, b]$. Since all the points of an interval lie between the endpoints (i.e., the pessimistic and optimistic values), it is reasonable that the interval values are replaced by the pessimistic and optimistic values. Each sample DMU in the constraint condition is divided into two corresponding sample DMUs based on the pessimistic and optimistic values. The process is given in Table 3, and the evaluated DMU is located in the fourth sample standard in the next year.

**Table 3:** **The processes of evaluation**

**Step**	**Standard**	**Objective value**	**Result**
1	[*k*/2]=2	>1	efficient
2	[(*k* + 2)/2]=3	>1	efficient
3	[(*k* + 3)/2]=4	[0.9117,1.953]	weak efficient

### The second example

Strategic groups are always used in the strategic management of insurance companies, and groups companies have similar business models or similar combinations of strategies. An insurance company can ascertain major competitors, obtain the competitive situation, and then formulate production strategy by analyzing strategic groups [55].

#### Dividing the sample insurance companies into ordered strategic groups

In this example, we only study the property insurance companies. The Appendix gives the overall production status of sample property insurance companies from 2010 to 2014 by the averaging method (the data is collected from Yearbooks of China’s Insurance). We assume that the formation of strategic groups is determined according to the following five manufacturing parameters: total number of employees (TNE), fixed assets (FA), sales tax and extra charges (STEC), earned insurance premiums (EIP), and expenses of payments (EP). The first three manufacturing parameters are inputs, and the others are outputs. FA, STEC, EIP, and EP are described in the unit of million Yuan RMB. As shown in Table 4, the sample DMUs are divided into six standards (i.e., six sample strategic groups).

**Table 4:** **Six strategic groups (from low to high)**

**Standard**	**Property Insurance Companies**
1	Bohai Property Insurance Company Limited; Chang an Property and liability Insurance Limited; Du-bang Property & Casualty Insurance Company Limited; Nipponkoa Insurance Company (China) Limited; China Continent Insurance; Ancheng Property & Casualty Insurance Company Limited.
2	Sinosafe Insurance Company Limited; Da Zhong Insurance Company Limited; Ming An Property & Casualty Insurance Company Limited; China Huanong Property & Casualty Insurance Company Limited; Liberty Insurance Company Limited.
3	Huatai Insurance Company of China, Limited; Aioi Insurance Company Limited; American Chubb Group of Insurance; Bank of China Insurance Company Limited; Zurish Insurance, Beijing; Alltrust Insurance Company Limited.
4	China Life Property & Casualty Insurance Company Limited; Tianan Property Insurance Company Limited; Dinghe Insurance; Sun Alliance Insurance Company; Generali China Insurance Company Limited; Sompo Japan Insurance (China) Company Limited.
5	The Tokio Marine & Nichido Fire Insurance Company (China) Limited; Anxin Agricultural Insurance Company Limited; AIG General Insurance Company China Limited; Allianz Insurance Company Guangzhou Branch; Hyundai Insurance (China) Company Limited.
6	Mitsui Sumitomo Insurance (China) Company Limited; Guoyuan Agricultural Insurance Company; China Export & Credit Insurance Corporation; Sunlight Mutual Insurance Company.

#### Predicting production status of Samsung Fire & Marine Insurance (China) Company Ltd

In this example, the evaluated DMU is Samsung Fire & Marine Insurance (China) Company Ltd. (Samsung F&M). The data of inputs and outputs are shown in Table 5. Based on historical production status from 2008 to 2013, the predicted production status of 2014 is given in Table 6. It is worth noting that Samsung F&M was established from 2006 in China, and thus the data of production status are limited.

**Table 5:** **The production status of Samsung F&M from 2008-2014**

**Year**	**TNE**	**FA**	**STEC**	**EIP**	**EP**
2008	62	1.67	2.28	65.78	55.68
2009	75	2.25	2.95	92.22	81.66
2010	91	2.43	9.2	87.94	68.42
2011	138	9.16	13.75	125.03	233.71
2012	192	10.01	19.88	134.23	102.67
2013	226	17.52	24.8	142.87	247.14
2014	325	19.27	28.1	195.56	217.11

**Table 6:** **The predicted production status in 2014**

**2014**	**TNE**	**FA**	**STEC**	**EIP**	**EP**
Predicted	286	20.03	29.8	174.2	232.5

#### Evaluating the production efficiency by the sample strategic groups

The predicted and actual production status of Samsung F&M in 2014 is evaluated by the ordered strategic groups. The process and results are shown in Table 7. Through comparison between the predicted strategic group and actual strategic group, we can see that the predicted results coincide with the actual results. Samsung F&M has similar efficiency to the fifth strategic group in 2014.

**Table 7:** **The evaluation processes and results**

**Step**	**Using predicted production status**			**Using actual production status**		
	**Standard**	**Objective values**	**Strategic Groups**	**Standard**	**Objective values**	**Strategic Groups**
1	[6/2]=3	>1	No	[6/2]=3	>1	No
2	[(6 + 2)/2]=4	>1	No	[(6 + 2)/2]=4	>1	No
3	[(6 + 4)/2]=5	[0.62,2.69]	Yes	[(6 + 4)/2]=5	[0.73,2.21]	Yes

## Conclusions

In the conventional DEA model, the inputs and outputs are known exactly, and the constraint condition consists of the evaluated DMUs. However, in many real applications, the observed data of the evaluated DMUs are variable over time. The efficiency of every evaluated DMU in a particular period may not be contrasted with the evaluated DMUs, but with sample standards determined by production strategy. Moreover, the development trend of the evaluated DMU, which is an important index to the budgetary decision-making and management system, is often required to be predicted.

In this paper, we proposed an extended DEA model to evaluate the efficiency of DMUs with historical observed data of inputs and outputs. Firstly, based on the historical observed data, we introduced the time series method to analyze and predict the development trend of the evaluated DMUs. Secondly, in the proposed model, there are many sample DMUs, which are divided into several ordered sample standards in terms of manufacturing parameters, and the constraint condition consists of one of the sample standards. Finally, we employ the algorithm based on a binary search tree to determine the constraint condition in order to reduce the computation complexity. One of the most intriguing and appealing points mentioned is that the paper is suitable for the decision-making, whether the evaluated DMUs are hospitals, universities, branches of a bank, or whatever.

## Appendix

**Table 8:** **The overall production status of sample property insurance companies. Unit: Person; RMB1000000**

**Property Insurance Companies**	**TNE**	**FA**	**STEC**	**EIP**	**EP**
Bank of China Insurance Company Limited	2,532	491.15	179.04	2,568.46	1,289.60
Aioi Insurance Company Limited	81	2.94	2.12	50.08	21.61
Ancheng Property & Casualty Insurance Company Limited	2,607	131.70	87.62	1,462.89	865.90
Allianz Insurance Company Guangzhou Branch	120	2.38	23.17	98.29	125.06
Anxin Agricultural Insurance Company Limited	362	171.66	25.61	637.41	378.86
Bohai Property Insurance Company Limited	3,254	242.34	85.02	1,268.91	822.27
China Continent Insurance	34,719	1,282.01	487.41	13,105.77	4,838.26
Da Zhong Insurance Company Limited	1,971	101.92	88.73	1,322.05	923.86
Dinghe Insurance	869	54.78	63.13	890.89	492.32
The Tokio Marine & Nichido Fire Insurance Company (China) Limited	280	9.30	23.02	402.39	207.36
Du-bang Property & Casualty Insurance Company Limited	8,663	599.14	104.56	3,378.89	1,070.38
China Life Property & Casualty Insurance Company Limited	14,576	690.25	1,023.03	14,584.57	8,087.69
Guoyuan Agricultural Insurance Company	1,023	50.97	13.90	1,428.10	954.51
Sinosafe Insurance Company Limited	5,835	1,167.15	286.24	4,370.28	2,043.66
China Huanong Property & Casualty Insurance Company Limited	370	10.86	14.85	230.85	136.24
Huatai Insurance Company of China, Limited	4,128	147.70	229.09	3,251.63	1,882.15
Liberty Insurance Company Limited	632	20.37	29.26	439.49	263.19
AIG General Insurance Company China Limited	931	9.27	27.98	749.04	281.25
Ming An Property & Casualty Insurance Company Limited	3,854	70.40	106.16	1,556.03	923.78
American Chubb Group of Insurance	122	4.14	7.25	99.86	49.87
Sompo Japan Insurance (China) Company Limited	307	9.78	12.71	296.89	166.82
Nipponkoa Insurance Company (China) Limited	47	1.85	1.72	23.67	13.97
Mitsui Sumitomo Insurance (China) Company Limited	319	4.84	22.23	567.87	333.88
Zurish Insurance, Beijing	74	1.35	13.10	70.46	41.40
Sun Alliance Insurance Company	94	2.44	8.05	95.44	56.85
Tianan Property Insurance Company Limited	14,084	221.65	453.61	6,870.18	4,848.25
Hyundai Insurance (China) Company Limited	53	4.14	4.76	57.60	78.41
Sunlight Mutual Insurance Company	2,170	102.14	16.71	1,846.27	1,283.38
Alltrust Insurance Company Limited	4,964	93.89	292.19	3,812.56	2,636.82
Chang an Property and liability Insurance Limited	3,316	139.71	93.77	1,276.34	876.85
China Export & Credit Insurance Corporation	2,159	395.69	38.97	4,101.75	4,958.59
Generali China Insurance Company Limited	137	5.05	10.33	144.06	61.92
